# Mithramycin delivery systems to develop effective therapies in sarcomas

**DOI:** 10.1186/s12951-021-01008-x

**Published:** 2021-09-06

**Authors:** Óscar Estupiñán, Enrique Niza, Iván Bravo, Verónica Rey, Juan Tornín, Borja Gallego, Pilar Clemente-Casares, Francisco Moris, Alberto Ocaña, Verónica Blanco-Lorenzo, Mar Rodríguez-Santamaría, Aitana Vallina-Álvarez, M. Victoria González, Aida Rodríguez, Daniel Hermida-Merino, Carlos Alonso-Moreno, René Rodríguez

**Affiliations:** 1 Sarcomas and Experimental Therapeutics Laboratory, Instituto de Investigación Sanitaria del Principado de Asturias (ISPA), Hospital Universitario Central de Asturias, Avenida de Roma, s/n, 33011 Oviedo, Spain; 2 Instituto Universitario de Oncología del Principado de Asturias, 33011 Oviedo, Spain; 3 CIBER en Oncología (CIBERONC), 28029 Madrid, Spain; 4 Centro Regional de Investigaciones Biomédicas, Unidad NanoCRIB, 02008 Albacete, Spain; 5 Universidad de Castilla-La Mancha, Facultad de Farmacia de Albacete, 02008 Albacete, Spain; 6 Materials Science and Engineering Department, Universitat Politècnica de Catalunya (UPC), Escola d’Enginyeria Barcelona Est (EEBE), 08019 Barcelona, Spain; 7 Institut de Recerca Sant Joan de Déu, 08034 Barcelona, Spain; 8 Centro Regional de Investigaciones Biomédicas (CRIB), UCLM, 02008 Albacete, Spain; 9 EntreChem SL, 33011 Oviedo, Spain; 10 Experimental Therapeutics Unit, Hospital Clínico San Carlos, IdISSC, 28040 Madrid, Spain; 11 Servicio de Anatomía Patológica, Hospital Universitario Central de Asturias, 33011 Oviedo, Spain; 12 Departamento de Cirugía, Universidad de Oviedo, 33006 Oviedo, Spain; 13 Netherlands Organisation for Scientific Research (NWO), DUBBLE@ESRF, 38000 Grenoble, France

**Keywords:** Mithramycin, Sarcoma, Soft tissue sarcoma, Chondrosarcoma, Polymeric nanoparticles, Polylactide, Small unilamellar vesicles liposomes, Hydrogels, Cancer stem cells

## Abstract

**Background:**

Sarcomas comprise a group of aggressive malignancies with very little treatment options beyond standard chemotherapy. Reposition of approved drugs represents an attractive approach to identify effective therapeutic compounds. One example is mithramycin (MTM), a natural antibiotic which has demonstrated a strong antitumour activity in several tumour types, including sarcomas. However, its widespread use in the clinic was limited by its poor toxicity profile.

**Results:**

In order to improve the therapeutic index of MTM, we have loaded MTM into newly developed nanocarrier formulations. First, polylactide (PLA) polymeric nanoparticles (NPs) were generated by nanoprecipitation. Also, liposomes (LIP) were prepared by ethanol injection and evaporation solvent method. Finally, MTM-loaded hydrogels (HG) were obtained by passive loading using a urea derivative non-peptidic hydrogelator. MTM-loaded NPs and LIP display optimal hydrodynamic radii between 80 and 105 nm with a very low polydispersity index (PdI) and encapsulation efficiencies (EE) of 92 and 30%, respectively. All formulations show a high stability and different release rates ranging from a fast release in HG (100% after 30 min) to more sustained release from NPs (100% after 24 h) and LIP (40% after 48 h). In vitro assays confirmed that all assayed MTM formulations retain the cytotoxic, anti-invasive and anti-stemness potential of free MTM in models of myxoid liposarcoma, undifferentiated pleomorphic sarcoma and chondrosarcoma. In addition, whole genome transcriptomic analysis evidenced the ability of MTM, both free and encapsulated, to act as a multi-repressor of several tumour-promoting pathways at once. Importantly, the treatment of mice bearing sarcoma xenografts showed that encapsulated MTM exhibited enhanced therapeutic effects and was better tolerated than free MTM.

**Conclusions:**

Overall, these novel formulations may represent an efficient and safer MTM-delivering alternative for sarcoma treatment.

**Graphical abstract:**

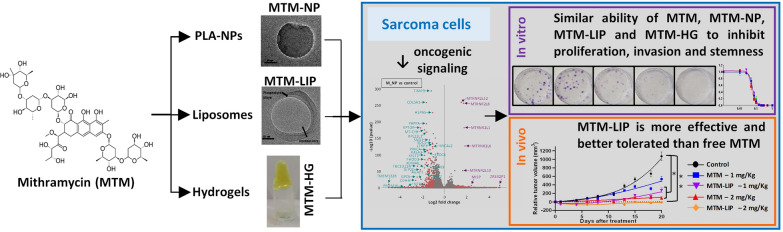

**Supplementary Information:**

The online version contains supplementary material available at 10.1186/s12951-021-01008-x.

## Introduction

Mithramycin (MTM), also called plycamicin is an antitumoural antibiotic natural product approved for the treatment of hypercalcemia [1] and which has also demonstrated good antitumour responses in the treatment of testicular cancer, glioblastoma or Ewing sarcoma [2–4]. Its mechanism of action is based on the binding to GC-rich sequences in DNA which, in turn, prevent the binding of transcription factors of the SP family in gene promoters [5, 6]. In addition, MTM has been found to interfere with the transcription mediated by a cancer-associated fusion gene [2, 7] and to induce differentiation through specific promoter reprogramming mechanisms [8]. However, despite these promising antitumour properties, the appearance of severe systemic toxicities, such as a dose-related bleeding syndrome and liver toxicity, has limited its clinical use [9, 10].

Two different strategies have been proposed to overcome the clinical limitation of MTM. The first one consists in the generation of structurally related analogues. In this regard, MTM derivatives have been proved to enhance antitumour activity and highly improve safety of MTM [11, 12]. The second strategy relies on the use of nanocarriers to reduce the delivery of the compound in non-tumoural areas, therefore increasing the therapeutic index. In addition, encapsulation of MTM could improve its pharmacokinetic and biodistribution profile [13]. MTM is characterized by a hydrophobic core and an outer hydrophilic shell which clearly limits the encapsulation. MTM has been encapsulated in large vesicles with a drug encapsulation efficiency of 60% for the first time [14]. At a later stage, polymeric nanoparticles were used to entrap MTM [15–17] including polymeric micelles manufactured by a new microfluidic approach. The MTM-loaded nanocarriers enhanced activity and exhibited lower toxicity when compared to the free drug in a study performed for beta-thalassemia [15]. Since MTM had shown promising results for the treatment of pancreatic carcinoma through the inhibition of the transcription factor Sp1, MTM were loaded into polymeric nanoparticles. These nanocarriers showed highly therapeutic efficacy in in vivo models of pancreatic carcinoma [17]. Similarly, MTM analogues-loaded polymeric nanoparticle formulations were reported to suppress lung cancer cells effectively [18].

Sarcomas comprise a group of aggressive malignancies which arise from transformed mesenchymal stromal/stem cells (MSCs) and/or their derived progenitor cells [19, 20]. Despite advances in the clinical management of these diseases, advanced sarcomas often show resistance to cytotoxic drugs such as doxorubicin, cisplatin or ifosfamide, which remain the first therapeutic option [21]. Among the factors contributing to this resistance, it is known that the acquisition of a stem-like phenotype by a subset of tumour cells, the so-called cancer stem cells (CSCs), play a relevant role in the outgrowing of drug-resistant clones and therefore, there is a need for therapies able to target these subpopulations [22].

In sarcomas, both MTM and MTM analogues have demonstrated a promising antitumour activity in Ewing sarcoma models through the specific inhibition of EWS-FLI1-mediated transcription [2, 8]. However, a phase I/II trial conducted to determine the dose-limiting toxicities, pharmacokinetics and activity of MTM in Ewing sarcoma concluded that MTM hepatotoxicity limited its administration at the minimal dose to potentially achieve clinical activity [23]. Later, the discovery of new mechanisms of action and synthetic lethalities in Ewing sarcoma have renewed interest in MTM [2, 24]. In addition, MTM analogues have demonstrated potent antitumour activity in MSC-based models of myxoid liposarcoma and undifferentiated pleomorphic sarcoma as well as in several primary cell lines of different types of sarcoma [12, 25]. Moreover, several groups have evidenced the great capacity of MTM and its derived analogues to target CSCs and inhibit stemness in sarcomas and other types of tumours [12, 25–29].

For this work, formulation of MTM-loaded hydrogel, liposomes and polymeric nanoparticles were developed with the aim of enhancing the therapeutic efficacy of MTM for the treatment of sarcoma. The polymeric nanoparticles were based on the FDA-approved Poly(rac-lactide) (PLA), liposomes (LIP) composed of zwitterionic phosphatidylcholine, mygliol and pluronic F-127 were the choice to encapsulate MTM in liposomal formulations, and a urea derivative non-peptidic hydrogelator was the platform to encapsulate MTM in a hydrogel [30]. The different nanoplatforms were assessed in terms of encapsulation and loading efficiency, release and stability. Furthermore, by using MSC-based and patient-derived models of sarcoma in in vitro assays, we demonstrated that all assayed MTM formulations retain the cytotoxic, anti-invasive and anti-stemness potential of free MTM. More relevantly, the treatment of sarcoma xenografts showed that encapsulated MTM is more efficient and tolerable than equivalent doses of free MTM. Additionally, transcriptomic analysis confirmed the ability of encapsulated MTM to repress relevant cancer-related pathways in sarcoma cells. Overall, these MTM delivery systems may represent useful alternatives to reposition MTM as a safe antitumour drug.

## Results

### Formulation, characterization, and stability of platforms

**Fig. 1:**
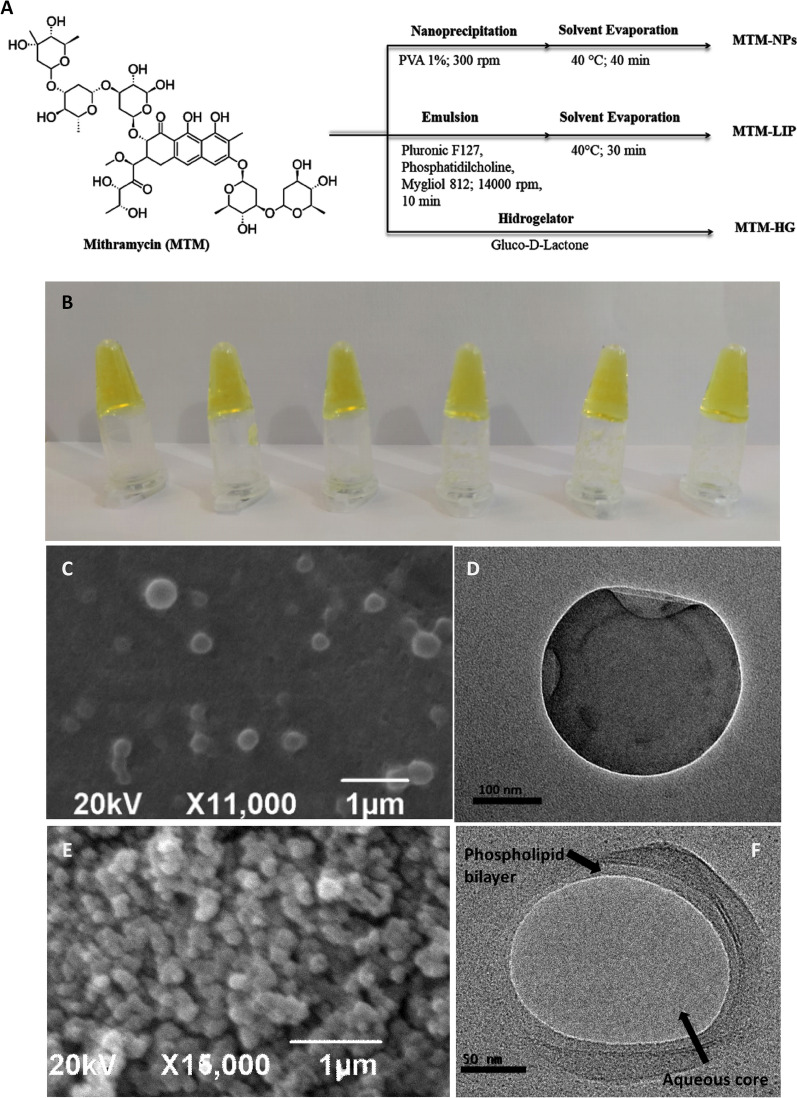
Formulation and morphology of MTM-NPs, MTM-LIP and MTM-HG. **A** Schematic formulation all nanodevices. **B** Image of MTM-HG. **C** SEM image of MTM-NPs (scale bar = 1 μm). **D** TEM image of MTM-NPs (scale bar = 100 nm). **E** SEM image of MTM-LIP (scale bar = 1 μm). **F** TEM image of MTM-LIP (scale bar = 100 nm)

MTM-loaded polymeric nanoparticles (MTM-NPs) were generated by the nanoprecipitation method [31] (Fig. 1A). MTM-loaded liposomal formulations (MTM-LIP) were prepared by ethanol injection and evaporation solvent method [32, 33]. MTM-to-lipid ratio of 1:5 w/w and a temperature of 40 °C were used for MTM encapsulation (Fig. 1A). MTM-loaded hydrogels (MTM-HG) were obtained by passive loading after slow pH change of the hydrogelator 5-(3-(4-nitrophenyl)ureido)isophthalic acid generated by the well-controlled hydrolysis of glucono-δ-lactone (GL) (Fig. 1A–B) [30]. MTM displays the capability of interaction with the hydrogel network by π-π stacking and the possibility of forming hydrogen bonds due to the presence of –OH groups in its chemical structure. MTM uptake was monitored by UV-visible spectroscopy.

Table 1 displays average size, polydispersity index (PdI), Z-potential, encapsulation (EE%) and loading efficiency (LE%) of liposomal, polymeric formulations, and the blank formulations. The non-loaded and MTM-loaded nanocarriers had a hydrodynamic radius (R_H_) close to 80 nm with a very low PdI. The Z-potential of the nanoplatforms showed high physical colloidal stability. Also, the LE and EE of MTM-NPs and MTM-LIP were calculated to be 3.1% ± 1.7% and 22.7% ± 2.5%, and 35.0% ± 0.4% and 92.5% ± 1.5, respectively. The particle size of MTM-NPs was observed by Scanning Electron Microscopy (SEM) with a diameter of 311 ± 10 nm as measured with Jeol image acquisition software (Fig. 1C), and spherical morphology as shown in the corresponding transmission electron microscopy (TEM) image (Fig. 1D). SEM and TEM showed globular liposomes and the aqueous core and the phospholipid bilayer could be identified (Fig. 1E**–**F).

**Table 1:** Characterization of MTM-loaded nanodevices

Formulation	R_H_, (nm)	PDI	Z-Potential (mV)	EE%	LE%
MTM-NPs	158.9 ± 0.9	0.16 ± 0.1	−31.9 ± 0.9	22.7 ± 2.5	3.1 ± 1.7
MTM-LIP	81.1 ± 0.2	0.08 ± 0.1	−27.6 ± 0.9	92.49 ± 1.5	35 ± 0.4
NPs	75.5 ± 0.5	0.1 ± 0.1	−24.5 ± 0.5	–	–
LIP	86.9 ± 0.8	0.1 ± 0.1	−17.1 ± 1.7	–	–

Hydrodynamic radius (R_H_), polydispersity index (PdI), Z-potential, encapsulation efficiency (EE%), and loading efficiency (LE%) of the formulations. Errors are 2σ

**Fig. 2:**
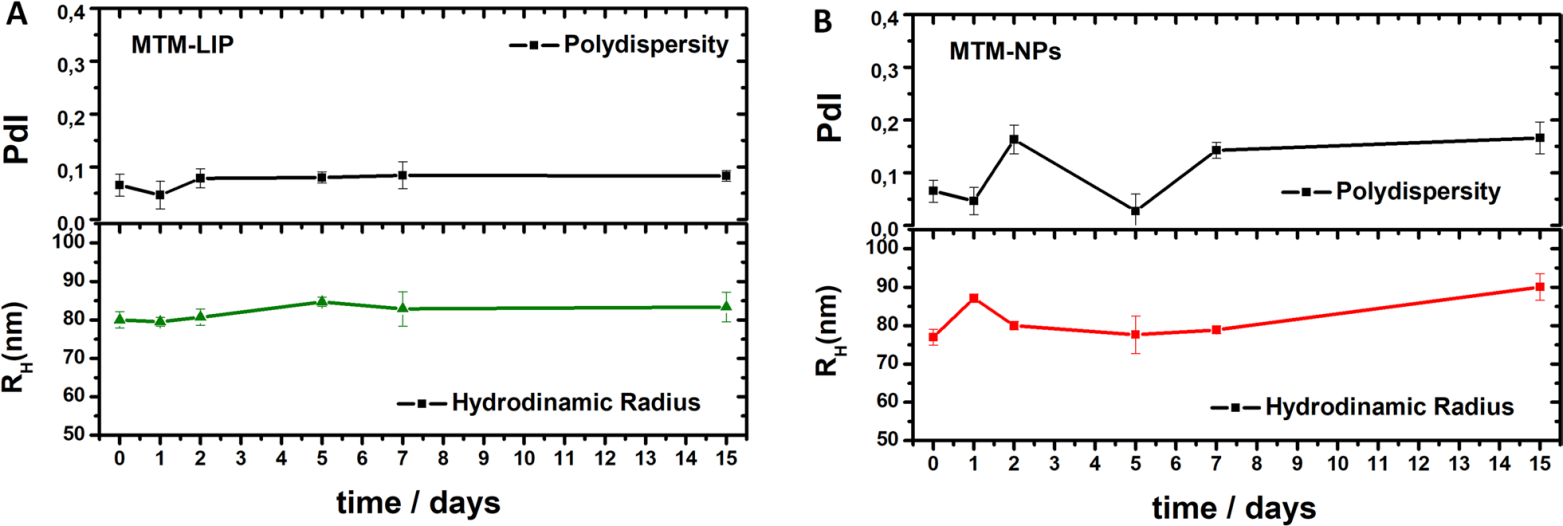
Storage stability of MTM-loaded nanodevices. **A**–**B** DLS analysis showing the stability of MTM-LIP (**A**) and MTM-NPs (**B**) nanoplatforms in PBS (pH 7.4). Data are expressed as mean ± SEM from at least three independent experiments

The storage stability of the formulations over time was studied in phosphate buffer saline (PBS). The values of R_H_ (nm) and PdI of the MTM-LIP and MTM-NPs were monitored by dynamic light scattering (DLS) at room temperature (Fig. 2A–B). Negligible increase in either particle size or PdI during a 7-day long experiment denoted high stability against aggregation for both formulations. The stability of the MTM-LIP was also performed in 10% human blood plasma (Additional file 1: Figure S1). The negligible changes of the liposomes suggested again a high stability against aggregation, this time in biological media. The slight increase in R_H_ during the first days can be related to the adsorption of a protein monolayer [34].

### Release studies

**Fig. 3:**
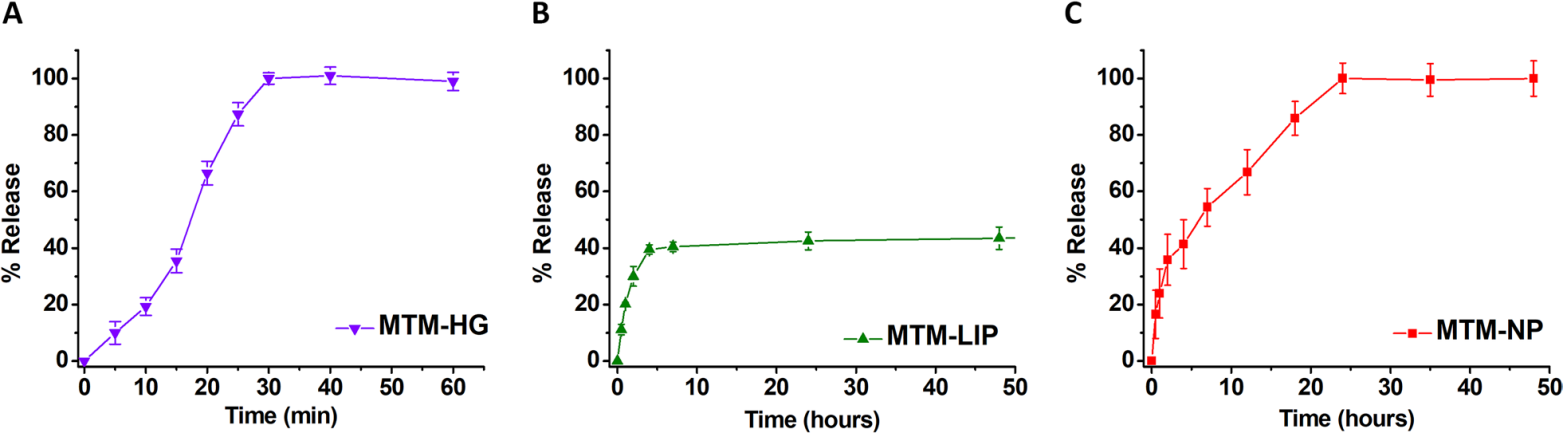
In vitro release profiles. Release kinetics MTM-HG (**A**), MTM-LIP (**B**) and MTM-NPs (**C**) in PBS (pH 7.4) at 37 ºC

In vitro MTM release from each formulation was investigated in pH 7.4 PBS at 37 °C. As shown in Fig. 3, different patterns were observed for each formulation. MTM-HG showed a two-stage release mechanism, a first release of the MTM molecules located at the surface followed by the MTM release from HG aggregates. In any case, drug release from HG is fast, with a full delivery of MTM after 30 min (Fig. 3A). In the case of MTM-LIP, a significant burst release was observed during the first 5 h followed by a slow release which did not exceed 40% after 50 h (Fig. 3B). In contrast, a relative fast release of MTM from MTM-NPs was observed in the initial 2 h. This initial phase was followed by a sustained release in which complete MTM delivery was achieved within 24 h (Fig. 3C).

### Antitumour effects of free and nanoencapsulated MTM in sarcoma cells

**Fig. 4:**
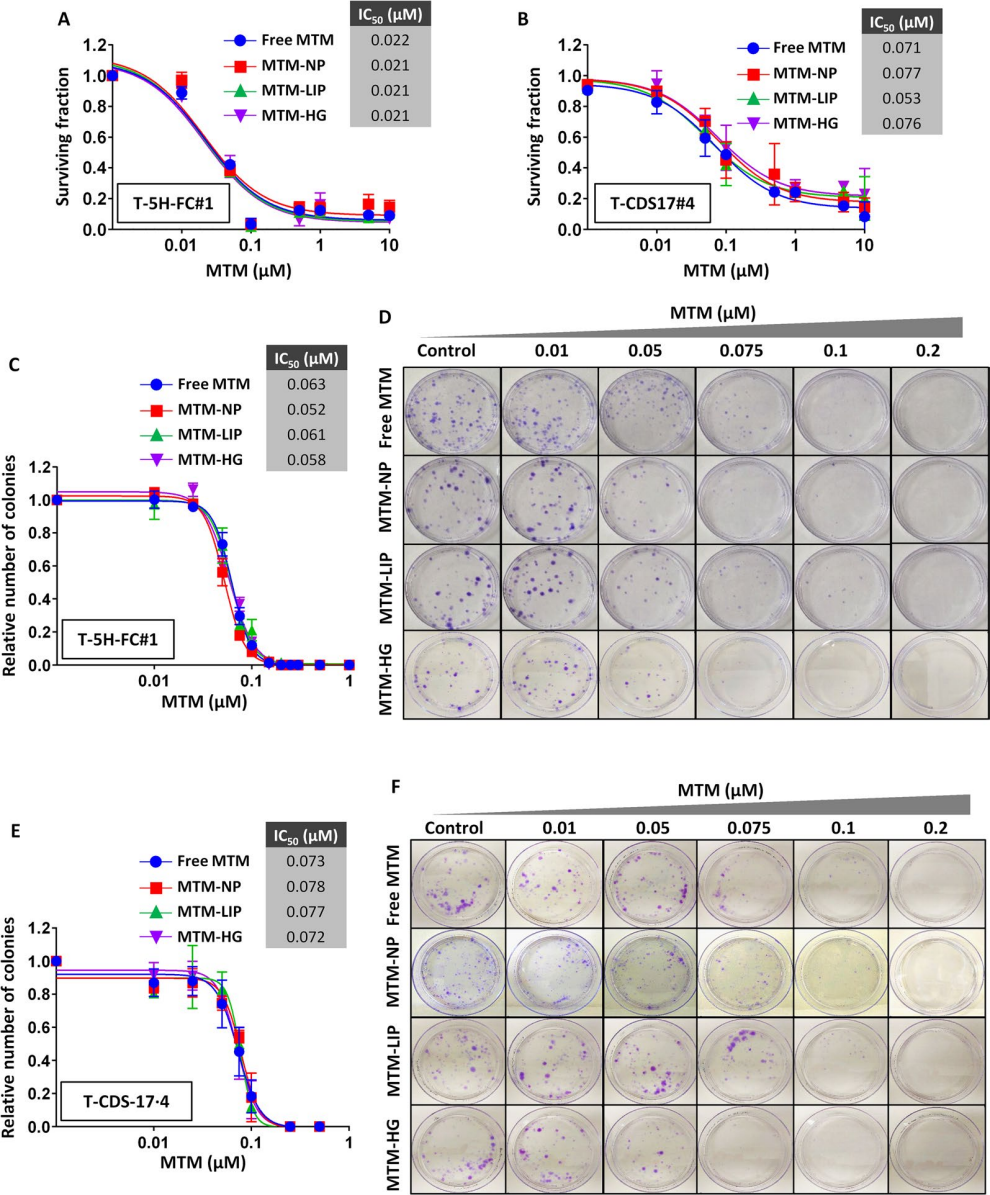
Antiproliferative effects induced by free and nanocarrier delivered MTM in sarcoma. **A–B** Cell viability (WST1 assay) measured after the treatment of T-5 H-FC#1 (**A**) and T-CDS17#4 (**B**) cells with increasing concentrations of free MTM or MTM loaded in polymeric nanoparticles (MTM-NPs), liposomes (MTM-LIP) and hydrogels (MTM-HG) for 72 h. IC_50_ values for each condition are shown. **C**–**F** Colony formation unit (CFU) assays in of T-5 H-FC#1 (**C**–**D**) and T-CDS17#4 (**E**–**F**) cells treated with increasing concentrations of the indicated MTM formulations for 24 h and left to form CFUs for 10 days. Summary graphics (**C** and **E**) and representative pictures of a colony formation assay (**D** and **F**) for each cell type are shown. Error bars represent the standard deviation of at least three independent experiments

In order to study the ability of different MTM formulations to target sarcoma cells, the induction of cell toxicity in dose-response cell survival assays was firstly evaluated. After 48-h treatments, the liposarcoma models MSC-5 H-FC and T-5 H-FC#1 displayed a similar response to free MTM and MTM-NPs, MTM-LIP and MTM-HG (Additional file 2: Figure S2A–B). These levels of antitumour activity in T-5 H-FC#1 cells were reproduced by MTM-NPs that were stored at 4ºC for 2 weeks, thus confirming the high stability of these formulations (Additional file 2: Figure S2C). Notably, empty devices of all formulations were unable to induce cell toxicity at any assayed concentration (Additional file 2: Figure S2A–B). As expected, MTM increased its antitumour activity after a longer treatment of 72 h in T-5 H-FC#1 cells, reaching IC_50_ values of approximately 20 nM for both free and nanocarrier delivered MTM (Fig. 4A). Likewise, T-CDS17#4 chondrosarcoma cells were also sensitive to nanomolar concentrations of MTM (IC_50_ ≈ 50–70 nM) and showed a similar response to all MTM formulations (Fig. 4B). Further exploring the antiproliferative effects of free and nanoencapsulated MTM, we also confirmed that all treatments showed a similar efficiency to target both T-5 H-FC#1 (Fig. 4C–D) and T-CDS17#4 (Fig. 4E–F) cells in colony-forming assays.

**Fig. 5:**
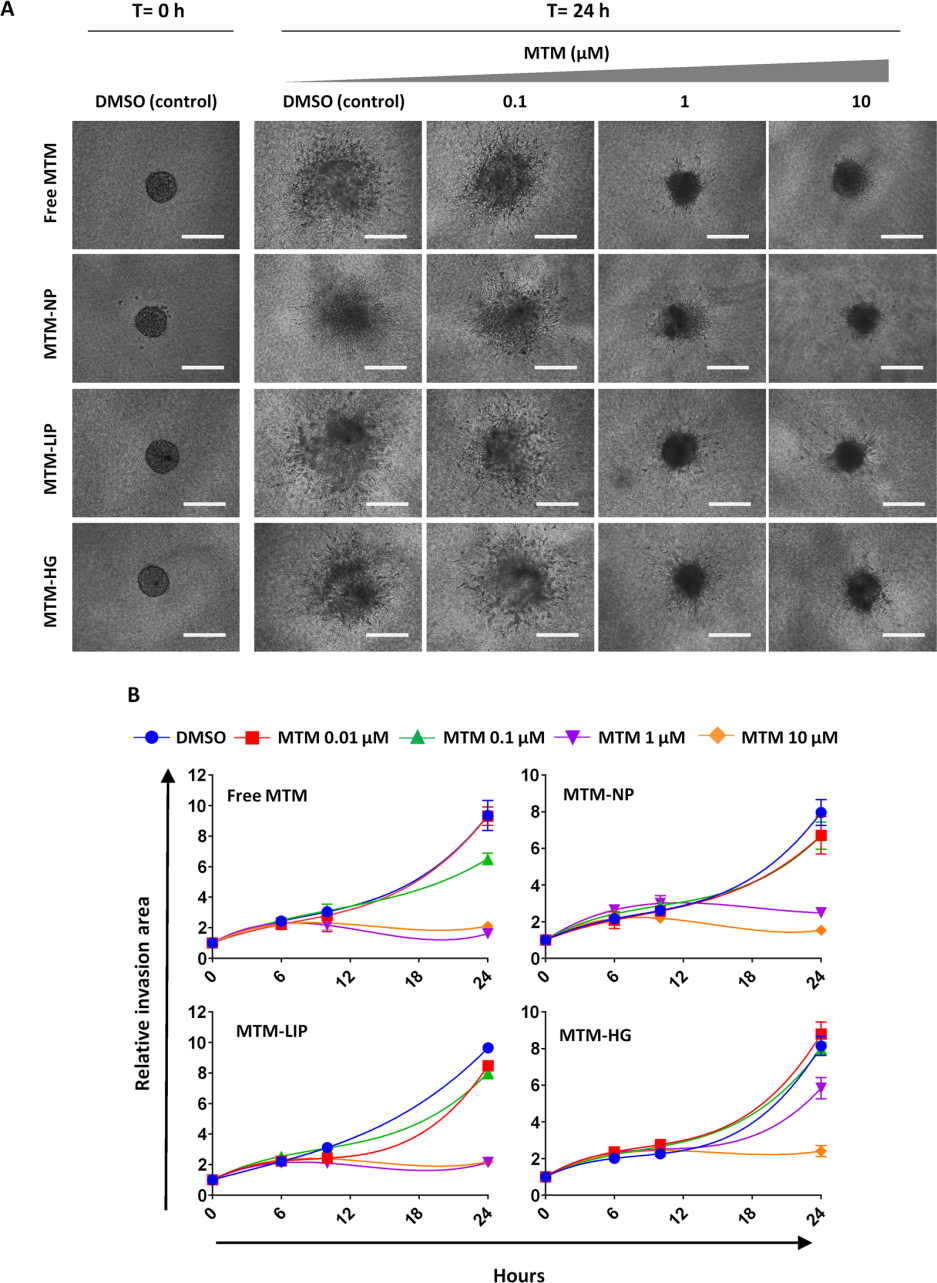
Effect of free and nanocarrier delivered MTM on cell invasion. **A**–**B** 3D spheroid invasion assays in T-5 H-FC#1 cells treated for 24 h with either DMSO (vehicle) or the indicated concentrations of the different MTM formulations. Representative images of spheroids at initial (t = 0) and final time (t = 24 h) (**A**) for the different treatments and the quantification of the invasive area at the indicated times (**B**) are displayed. Scale bars = 200 m. Data (mean and standard deviation) are calculated from at least 6 spheroids per condition and time point and expressed relative to DMSO-treated cells

Next, we analysed the effect of MTM formulations to alter the invasive properties of sarcoma cells. With this aim, we studied the ability of T-5 H-FC#1 spheroids to invade 3D collagen matrices. We found that concentrations of free MTM equal or higher than 1 µM were able to completely suppress cell invasion after 24 h. Similar results were found for MTM-NPs and MTM-LIP, whereas MTM-HG showed only a partial response after a 1 µM treatment (Fig. 5 and Additional file 3: Figure S3).

### Nanocarrier delivered MTM targets CSC subpopulations in sarcoma

**Fig. 6:**
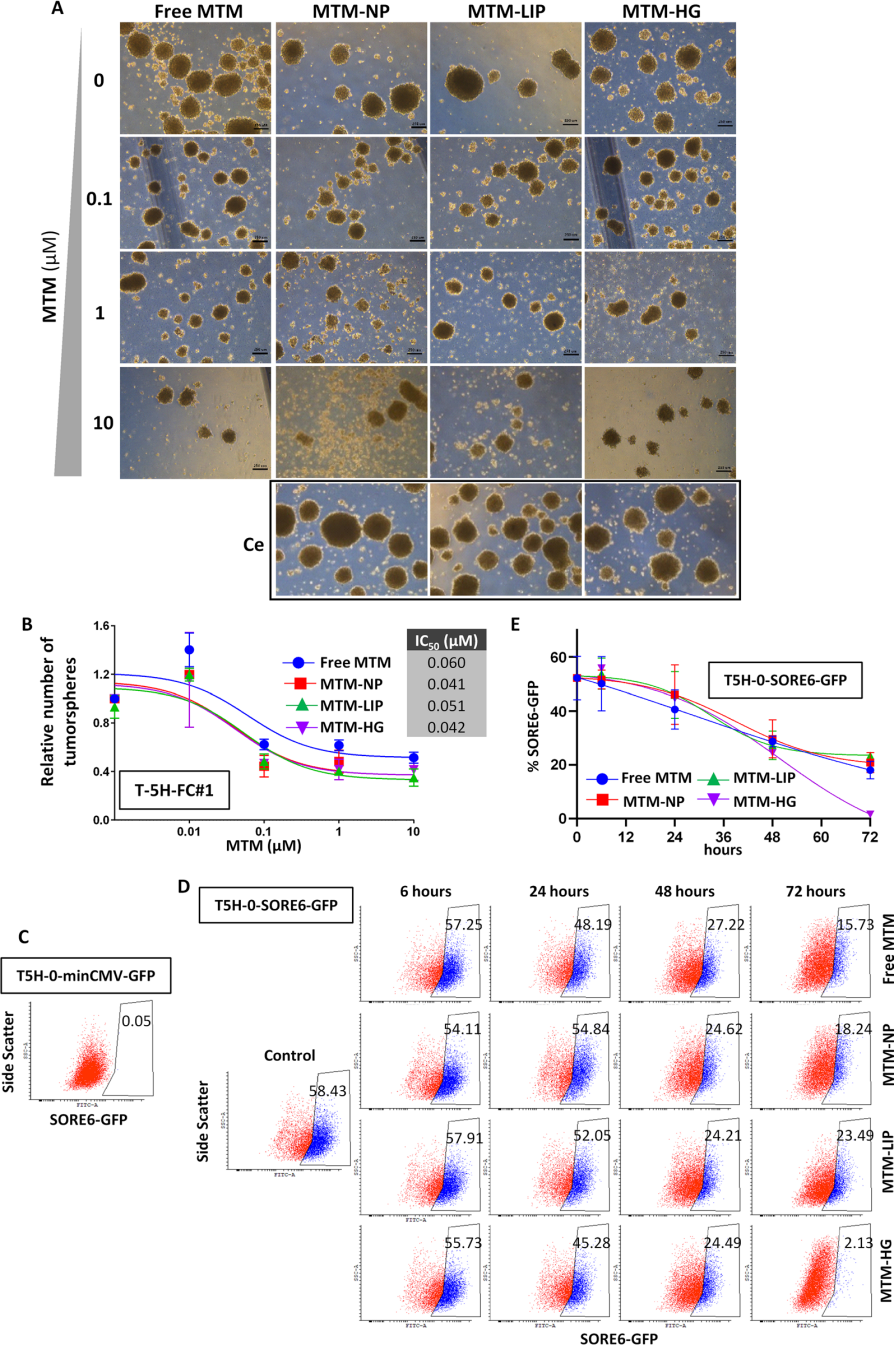
Effect of free and nanocarrier delivered MTM on CSC subpopulations. **A**–**B** CSC-enriched tumourspheres of T-5 H-FC#1 cells were treated with increased concentrations of the indicated MTM formulations for 72 h. Treatments with an amount of empty nanoparticles corresponding to 10 µM (Ce) were also included. Representative images of the spheres cultures (**A**) and the quantification of the spheres (represented as % of control) (**B**) remaining at the end of experiment are shown. Scale bars = 250 μm. Error bars represent the standard deviation of at least three independent experiments. **C**–**E** Analysis of the percentage of cells presenting transcriptional activity of the pluripotency factors SOX2 and OCT4 (SORE6 activity) after the treatment with free ree MTM, MTM-NPs, MTM-LIP or MTM-HG. **C**–**D** Representative flow cytometry analysis of the SORE6 + population in untreated T5H-O-minCMV-GFP (gating control) (**C**) and in T5H-O-SORE6-GFP cells treated for with 20 nM MTM for the indicated times (**D**). **C** Graph showing the mean and standard deviation of three independent experiments

MTM and its recently developed analogues have demonstrated their ability to target tumour subpopulations presenting CSC properties in several types of tumours, including sarcomas [12, 25–29]. Therefore, we studied the effect of MTM nano-encapsulation on its ability to inhibit the growth of CSC-enriched 3D clonal sphere cultures (tumourspheres) of T-5 H-FC#1 cells. Importantly, we had previously demonstrated that this tumoursphere model was highly enriched in CSCs as seen by their increased ability to initiate tumour growth in transplantation assays in immunocompromised mice [35]. Using this model, we found that both free MTM and all assayed MTM nanocarrier systems were similarly able to reduce the number (IC_50_ ≈ 40–60 nM) and the size of T-5 H-FC#1 tumourspheres, without observing any toxic effect after the treatment with empty nanoparticles (Fig. 6A–B).

To further confirm the ability of MTM formulations to target CSC subpopulations in sarcomas, we made use of a previously developed model of undifferentiated pleomorphic sarcoma (T-5 H-O cells) expressing a lentiviral reporter system which include a composite SOX2/OCT4 response element (SORE6) coupled to a cytomegalovirus promoter to drive the expression of the GFP reporter gene (T-5 H-O-SORE-GFP cells) [25, 36]. The SORE6 reporter allows the dynamic monitoring of CSC subpopulations based on transcriptional activity due to the pluripotency factors SOX2 and OCT4. In addition, this reporter has proven its efficacy to test the potential of antitumour drugs to target CSC subpopulations [25, 37]. In this system, cells carrying a control construction without SORE6 (T-5 H-O-minCMV-GFP cells) were used as gating controls in flow cytometry analyses (Fig. 6C and Additional file 4: Figure S4). To analyse the effect of MTM on CSC subpopulations, T-5 H-O-SORE-GFP cells were treated with IC_50_ concentrations of free MTM, MTM-NPs, MTM-LIP and MTM-HG in time-course experiments. As shown by flow cytometry analysis, all MTM formulations induced a gradual reduction of SORE6 + subpopulation that reached a 75–95% decrease after 72 h of treatment (Fig. 6D–E), thus confirming the ability of MTM, both delivered free or nanoencapsulated, to target CSCs in sarcomas.

Altogether, we found that all assayed MTM-loaded nanoparticles were able to closely mimic the anti-proliferative, anti-invasive and anti-stemness effects of free MTM in different sarcoma models.

### Gene expression profiling of MTA-treated sarcoma cells

**Fig. 7:**
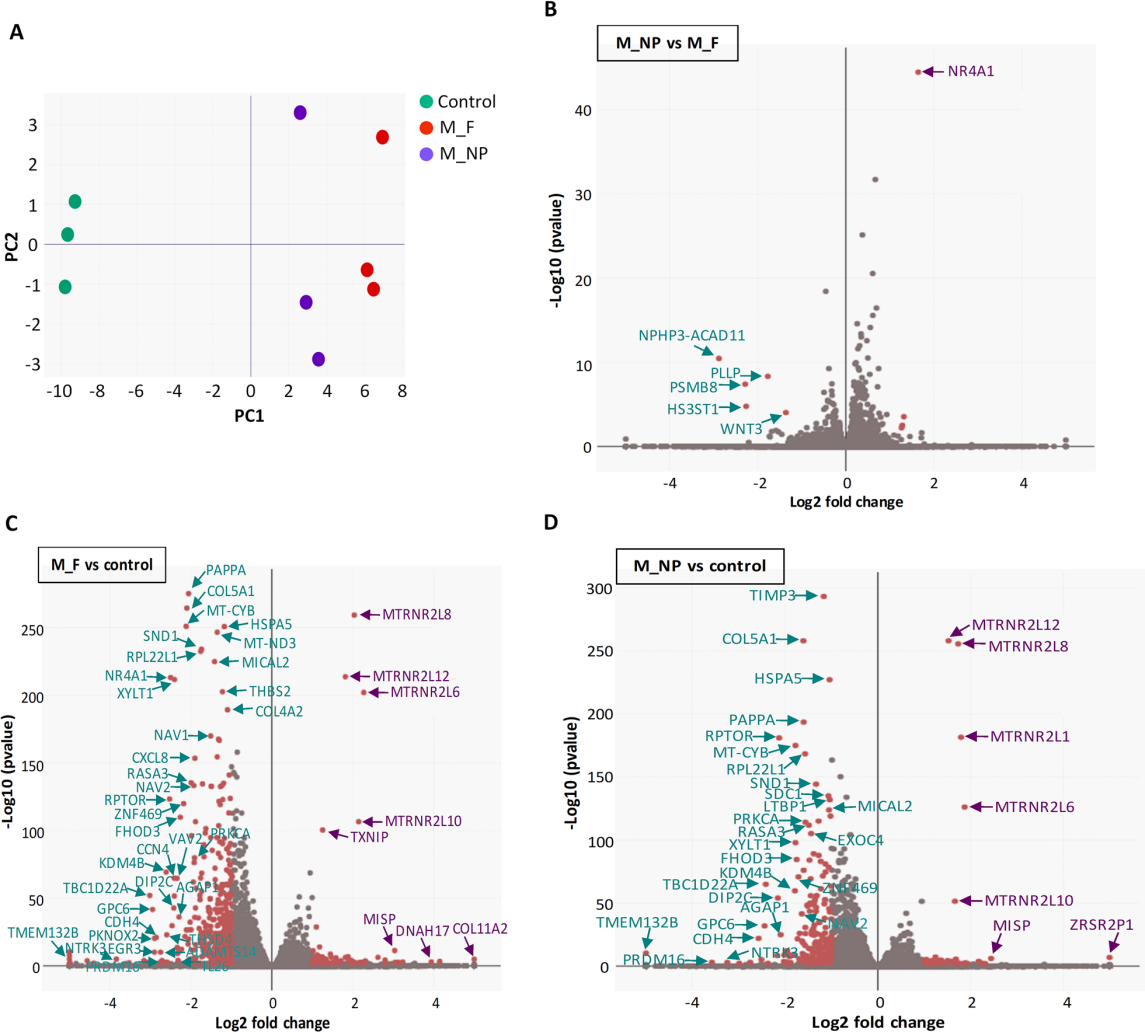
Transcriptome analysis of cells treated with free and nanocarrier delivered MTM. RNA seq analysis of T-5 H-FC#1 cells treated in triplicate with either DMSO (control), 25 nM free MTM (M_F) or 25 nM MTM-NPs (M_NP) for 24 h. **A** Principal component analysis of all samples. **B**–**D** Volcano plots showing those genes significantly up-regulated and downregulated (fold change ≤-2 or ≥ 2 and padj < 0.01; red dots) when comparing M_NP vs. M_F (**B**), M_F vs. control (**C**) and M_NP vs. control (**D**). Selected genes displaying highly significant *p* values and/or high fold change modulation are indicated

To gain insight into the mechanisms involved in the anti-tumour effects of MTM in sarcoma cells and analyse whether these molecular features could be influenced by nanoencapsulation, we performed RNAseq analyses of T-5 H-FC#1 cells treated with the IC_50_ values (25 nM) of free MTM and MTM-NPs or the drug vehicle for 24 h. First, we used the gene expression data obtained from triplicate samples of each condition to conduct a principal component (PC) analysis. Both free MTM and MTM-NPs treated samples group with close PC1 values but far from control samples (Fig. 7A). Then, we selected differentially expressed genes (DEG) (fold change ≤-2 or ≥ 2 and padj < 0.01) in MTM-NPs vs. free MTM, free MTM vs. control and MTM-NPs vs. control comparisons. In agreement, with the result of the PC analysis, the gene expression profiles of cells treated with MTM-NPs or free MTM were very similar, with only 5 downregulated and 4 upregulared DEGs between these conditions (Fig. 7B and Additional file 5: Table S1). In comparisons against the control condition, we found that free MTM induced a higher level of gene expression modulation (522 DEGs) (Fig. 7C and Additional file 6: Table S2) than MTM-NPs (242 DEGs) (Fig. 7D and Additional file 7: Table S3). In any case, as expected, there was a high degree of overlapping between genes regulated by both treatments (83% of DEGs in MTM-NPs treated cells were also modulated in free MTM-treated cells). Significantly, this analysis showed that MTM (whether free or encapsulated) induced a general repression of gene expression. Thus, 400 of the 522 DEGs in cells treated with free MTM (Fig. 7C and Additional file 6: Table S2) and 201 of the 242 DEGs in MTM-NPs treated cells (Fig. 7D and Additional file 7: Table S3) were downregulated.

**Fig. 8:**
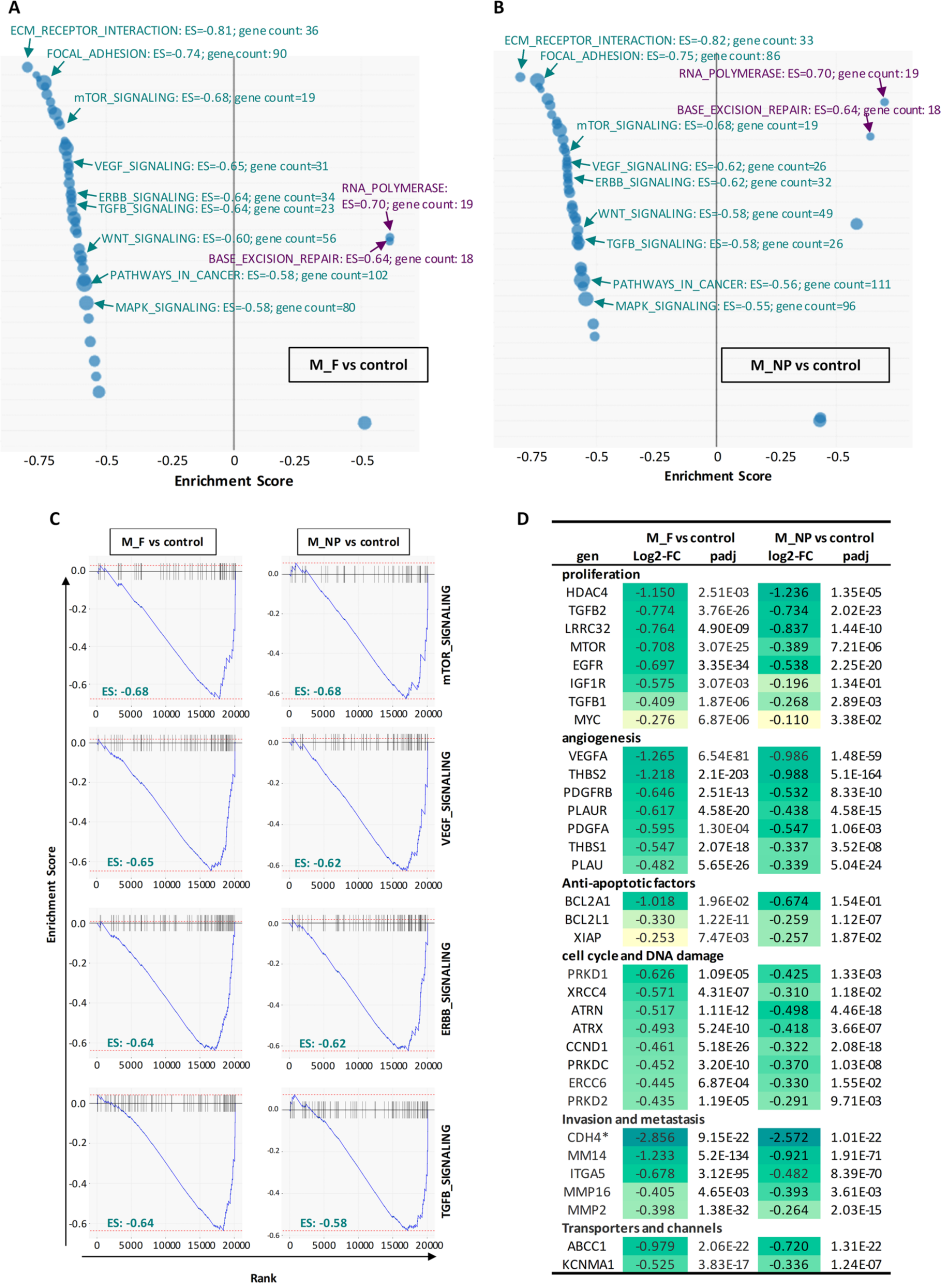
Signaling pathways altered by the treatment with free and nanocarrier delivered MTM. RNAseq data were used to perform gene ontology analyses. **A**–**B** KEGG pathway analysis showing those signaling routes significantly altered (enrichment score (ES) ≤-0.5 or ≥ 0.5 and padj < 0.01; blue circles) when comparing M_F vs. control (A) and M_NP vs. control (**B**). Circle diameter for each pathway reflect the number of genes involved in the pathway (gene count) showing altered expression. Information for relevant upregulated (purple text) of downregulated (green text) pathways is displayed. **C** GSEA analysis of selected signaling pathways in M_F vs. control (left) and M_NP vs. control (right) comparisons. **D** Fold change expression (expressed as Log2-FC) and padj values obtained in the indicated comparisons for a panel genes known to be regulated by SP1. (* Several cadherins have been described to be targets of SP1, although CDH4 has not been described yet as a SP1-regulated gene)

These common patterns of gene expression also resulted in the modulation of common signalling pathways by free MTM and MTM-NPs. Thus, KEGG pathways analyses of DEGs showed that 93% of altered signalling routes were mutually modulated by both treatments. In line with the gene expression downregulation observed after the treatments, most of these pathways (47 out of 50 altered pathways in MTM-NPs and 43 out of 46 in free MTM) displayed significant negative enrichment scores (ES < −0.5 and padj < 0.01) and were therefore predicted to became inhibited both in free MTM and MTM-NPs-treated cells (Fig. 8A–B, Additional files 8, 9: Tables S4 and S5). Among these commonly repressed pathways we found several routes that are frequently over-activated in cancer, such as ECM receptor interaction, focal adhesion, mTOR signaling, VEGF signaling, ERBB signaling, TGFβ signaling or WNT signaling (Fig. 7A–B). Likewise, Gene Set Enrichment Analysis (GSEA) confirmed the inactivation of these relevant signaling pathways after the treatment with both MTM formulations (Fig. 8C). On the other hand, only 3 pathways, related to RNA polymerase activity and base excision repair, were predicted to be activated after MTM treatment, regardless of free or encapsulated delivery of the drug (Fig. 8A–B).

**Fig. 9:**
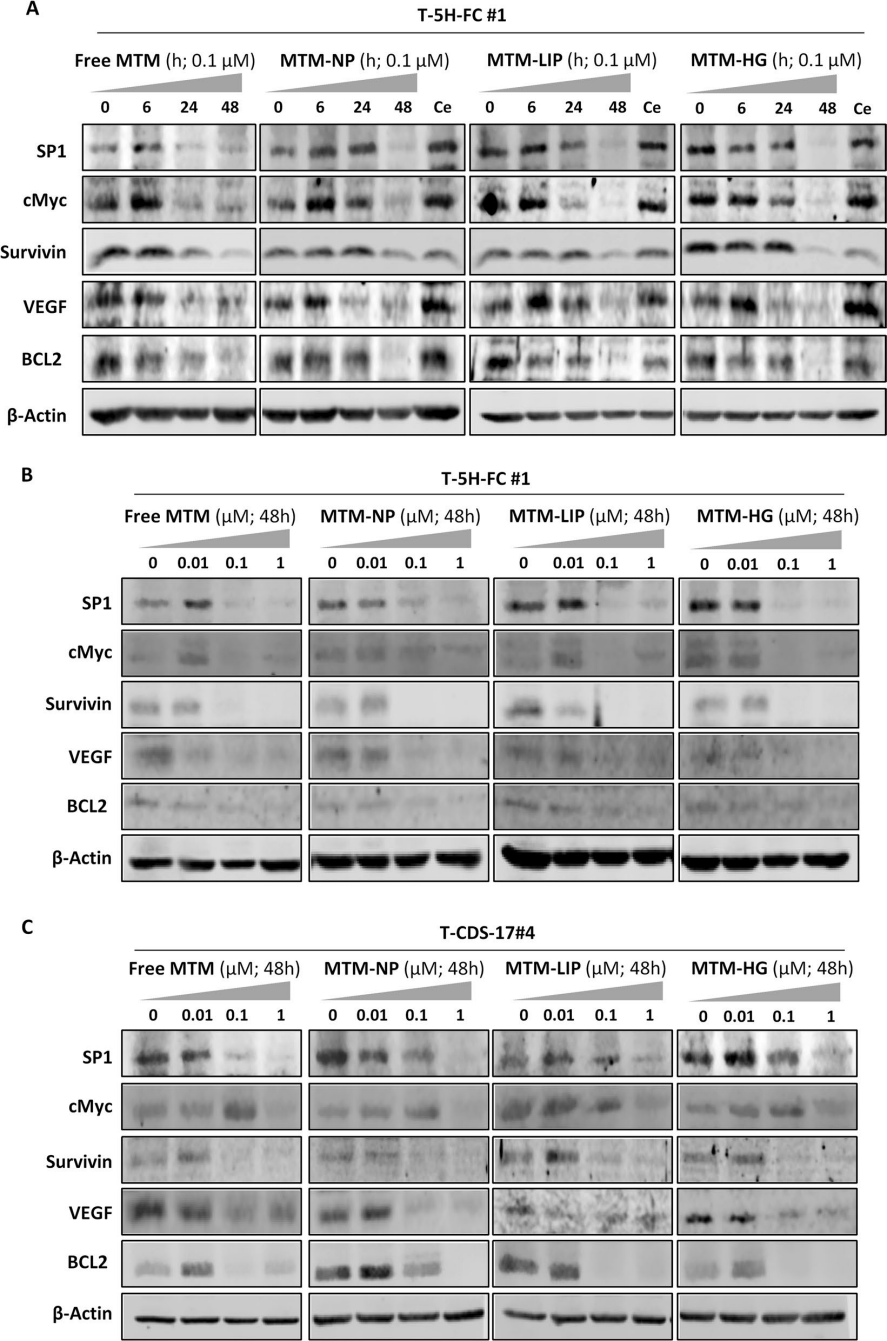
Inhibition of SP1 signaling by free and nanocarrier delivered MTM. **A** Western blotting analyses of SP1 and several SP1 downstream targets in T-5 H-FC#1 cells treated with 0.1 µM free MTA or MTA-loaded nanoparticles for the indicated times. A control of a 48 h-treatment with empty NPs (Ce) for each formulation is included. **B**–**C** Western blotting analyses of SP1-related factors in T-5 H-FC#1 (**B**) and T-CDS-17#4 cells (**C**) treated with the indicated concentrations of the different MTM formulations for 48 h. β-Actin levels were used as loading controls

One of the best described anti-tumour mechanisms of action for MTM is their ability to inhibit the expression of the pleiotropic SP1 transcription factor and its downstream signaling [5, 6]. Therefore, we compared the ability of free MTM and encapsulated MTM to inhibit the expression of SP1 and several well-known SP1 downstream targets [6, 38]. First, by using RNAseq data, we found a robust pattern of gene expression repression in a wide panel of SP1-modulated genes both in free MTM and MTM-NPs treated cells (Fig. 8D). Then, we confirmed at protein level that all MTM formulations were able to inhibit the expression of SP1 and its targets, c-MYC, Survivin, VEGF, and BCL2 (Fig. 9). In T-5 H-FC#1 cells, we observed an efficient time-course (Fig. 9A) and dose-response dependent (Fig. 9B) ability of MTM, irrespective of whether it was delivered free or encapsulated, to repress all analyzed targets. An MTM concentration of 0.1 µM was able to almost completely repress all targets after 48 h of treatment. Notably, no effect was observed after the treatment with empty nanoparticles (Fig. 9A). Likewise, all MTM formulations showed a similar dose-dependent ability to inhibit SP1 and SP1 targets in T-CDS-17#4 cells (Fig. 9C).

In sum, these results confirmed that MTM encapsulated in NPs, LIP and HG retain the ability of free MTM to repress relevant cancer-related pathways in sarcoma cells.

### In vivo antitumour activity of free and encapsulated MTM

**Fig. 10:**
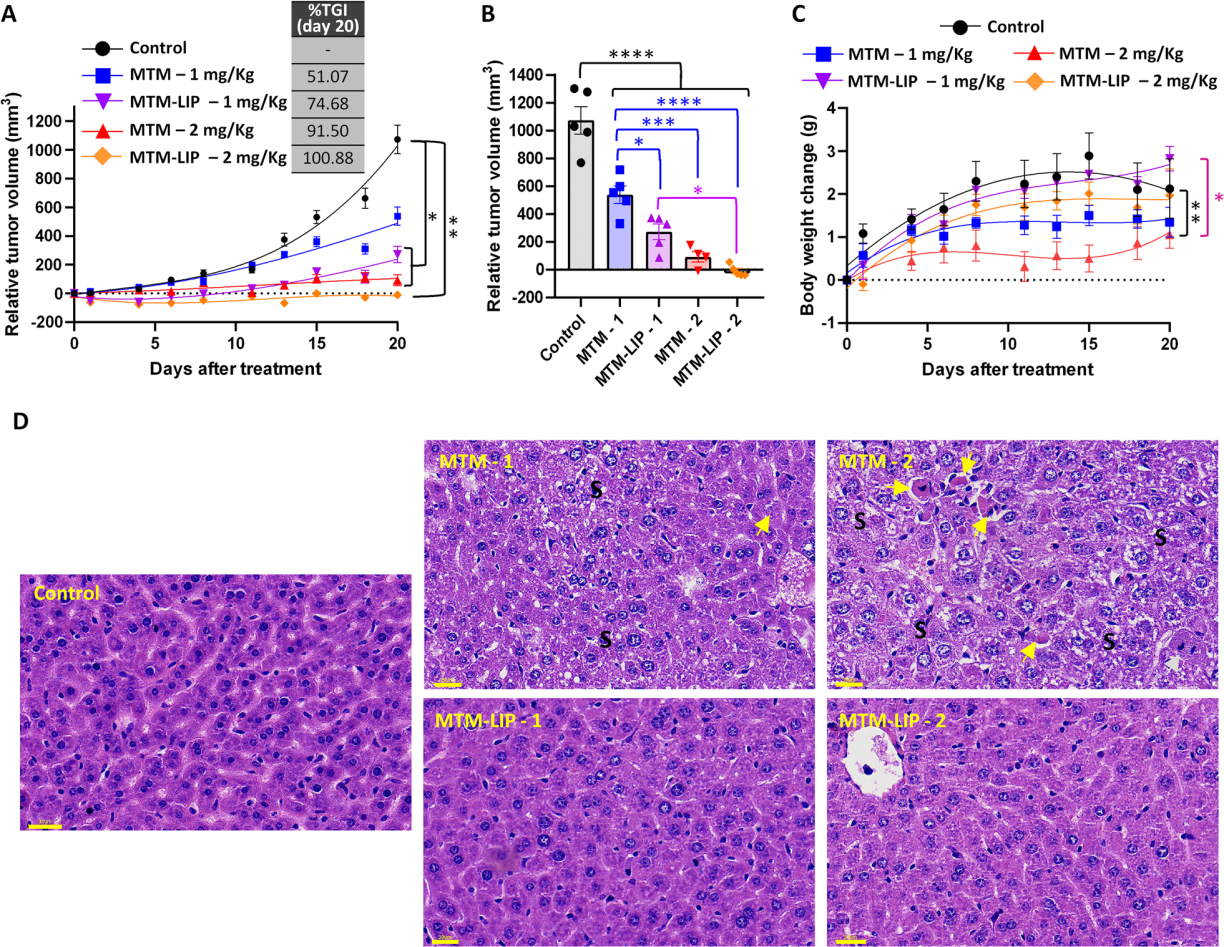
In vivo effect of free MTM and MTM-LIP. T-5 H-FC#1 established xenografts were randomly assigned to five different groups (n = 5 per group) and treated intravenous with vehicle (PBS, control), free MTM at 1 or 2 mg/kg or MTM-LIP at 1 or 2 mg/KG every 3–4 days (twice a week). **A** Curves representing the mean relative tumour volume of T-5 H-FC#1 xenografts during the treatments. Drug efficacy expressed as the percentage of TGI is indicated. **B** Distribution of tumour volumes at the end of the experiment (day 20 after the start of the treatment). **C** Change in the body weights of mice during the treatments. **D** H&E staining of formalin-fixed paraffin embedded livers extracted at the experimental end-point. Areas presenting microvesicular steatosis (S), necrotic cells (yellow arrows) and mitosis (grey arrows) are indicated. Scale bars = 30 μm. Error bars represent the SEM and asterisks indicate statistically significant differences between groups in one-way ANOVA Turkey’s tests (*:p < 0.05; **:p < 0.01; ***:p < 0.001; ****:p < 0.001)

Finally, to test whether the encapsulation of MTM may result in increased in vivo efficacy and safety of this drug, we treated immunodeficient mice carrying T-5 H-FC#1 cells with free MTM or MTM-LIP. Two different doses (1 and 2 mg/Kg) of MTM-LIP demonstrated a greater ability to reduce tumour growth than equivalent doses of free MTM. The 1 mg/Kg MTM-LIP group, but not the 1 mg/Kg free MTM, showed a significantly delayed tumour growth curve than the control series. Likewise, the difference between control and 2 mg/Kg MTM-LIP series was more significant than that observed between control and 2 mg/Kg free MTM groups (Fig. 10A). At the experimental end-point (day 20 after the start of treatment), we also found that tumours treated with 1 mg/Kg MTM-LIP were significantly smaller than those treated with an equal dose of free MTM (Fig. 10B). In addition, we observed more favorable values of TGI when mice were treated with MTM-LIP. Thus, TGI ranged from 51 to 74% for the groups treated with 1 mg/Kg of free MTM and MTM-LIP respectively and between 91 and 100% for groups treated with 2 mg/Kg of free MTM and MTM-LIP respectively (Fig. 10A).

We have not found deaths or loss of body weight in any of the groups; however, the groups treated with free MTM gained weight at lower rate than those treated with equivalent doses of MTM-LIP. Thus, the body weight change curve of mice treated with 2 mg/Kg free MTM, but not that of those treated with 2 mg/Kg MTM-LIP, was significantly different from the curve of control mice (Fig. 10C). In accordance with this observation, the histopathological examination of livers extracted from all the mice of the MTM−2 mg/Kg group showed a high level of lipid accumulation (microvesicular steatosis), accompanied by the presence of localized hepatocellular necrosis. Intriguingly, one of the samples of this group also presented an unusually high number of mitosis. This pattern of toxicity was also observed at lower levels in all livers of the MTM − 1 mg/Kg group. On the other hand, none of the livers from the MTM-LIP−1 mg/Kg showed signs of hepatotoxicity and only two of the samples from the MTM-LIP−2 mg/Kg group presented low levels of steatosis or occasional necrotic cells (Fig. 10D).

Taken together, this data indicate that MTM-LIP is more efficient and tolerable than equivalent doses of free MTM. Indeed, the treatment with 2 mg/Kg MTM-LIP caused tumour regression in the T-5 H-FC#1 sarcoma model without observing severe adverse effects in mice.

## Discussion

Over 15% of all pediatric solid malignant cancers are sarcomas [21]. The variety of subtypes of sarcomas and the rarity of the disease render them a very complex oncology entity to study. Very few effective and approved agents for the treatment of sarcomas exist. Chemotherapeutic drugs like doxorubicin or ifosfamide, alone or the combination thereof, constitute the main first-line systemic therapeutic option for patients suffering from sarcoma. In this context, the prognosis of advanced sarcoma patients is still unfavorable. Several promising drugs, including immunotherapy, kinase inhibitors or epigenetic modulators, among others, are therapeutic avenues currently undergoing study within ongoing clinical trials [21]. Among these strategies, the repurpose of drugs that have demonstrated anti-tumour efficacy in the past, also merits consideration. In this regard, MTM is a natural product approved by the FDA since 1970 s that has been used clinically for the treatment of testicular carcinoma, glioblastomas and Ewing sarcoma [2–4]. However, gastrointestinal, hepatic, kidney and bone toxicity limited its widespread clinical implementation [9, 10]. Several strategies have been followed to take advantage of MTM anti-tumour properties while improving its toxicity profile. First, several analogues of MTM, such as EC-8042, have been reported to enhance its pharmacological profile while retaining a strong anti-tumour effect in sarcomas and other types of tumours [6, 8, 11, 12, 37, 39]. On the other hand, the use of nanotechnology holds great promise for improving drug bioavailability minimizing toxicity from systemic exposure. The extended release of MTM from nanodevice systems could result in augmenting its therapeutic index, and improving several PK properties such as circulation time, metabolism or biodistribution. These facts may open the door for its evaluation in solid tumours.

The generation of MTM delivery systems is an interesting challenge due to its amphiphilic nature. MTM is highly soluble in water which causes its escape to the external aqueous phase, but it has a tricyclic aromatic polyketide moiety which gives it solubility to some extent in several organic solvents, including dimethylsulfoxide or methanol. In this sense, herein different devices were explored to entrap MTM. Polymeric NPs are widely used as carriers for lipophilic drugs, since they can avoid renal clearance, circulate in the body for a prolonged time and accumulate in the tumour through the enhanced permeability and retention effect. Hydrogels are drug delivery systems mainly designed for the entrapment of hydrophilic drugs, whereas liposomes are systems to encapsulate drugs in the aqueous reservoir (hydrophilic drugs) or entrap them in the lipid bilayer (hydrophobic drugs). PLA were used as raw material for the generation of the polymeric NPs since it is approved by the FDA and is biodegradable and biocompatible. The hydrogelator was selected for entrapping MTM because it possesses a significant low minimum gelation concentration and allow fine-tuning of pH. Finally, a liposomal MTM formulation is reported based on the MTM encapsulation efficiency and the physical and chemical stabilities obtained. To the best of our knowledge, PLA and HG have never been previously reported as raw materials for the generation of MTM delivery systems. As a proof of concept, we have very recently encapsulated MTM in poly(lactic-co-glycolic acid) (PLGA) micelles and transfersomes [40]. However, the unfavorable high size PLGA micelles and the high PDI transfersomes encourage us to explore other nanodevice platforms for encapsulation.

MTM-NPs were obtained by the nanoprecipitation method. PLA was dissolved in a semipolar water miscible organic solvent, such as acetone and the NPs were formed after injection of the PLA solution into an aqueous phase. This methodology produced MTM-NPS with an optimal size, low polydispersity and a prominent encapsulation efficiency. When compared to the MTM entrapment in PLGA NPs, similar values in terms of size, narrow size distribution, and Z-potential were obtained [16]. In other work, MTM-NPs obtained using a single-emulsion solvent evaporation method with pegylated PGLA and after filtration gave rise to smaller NPs of < 25 nm in average size [17]. Also using the emulsion solvent evaporation method, we previously generated MTM-PLGA micelles with a greater size of around 220 nm [40]. However, the micellization procedure can be unfavorable for drug delivery. The disassembly of micelles may cause the abrupt release of the drugs. Concerning the generation of MTM-HG, MTM was entrapped during the network fibrillation process in a passive loading and the gels were obtained by the slow acidification produced by the hydrolysis of GL [30]. The HG was stable at temperatures above the boiling point of water. Finally, in this work we successfully obtained liposomal formulations by the ethanol injection and evaporation solvent method and using miglyol and pluronic F-127 as surfactants. This NVs displayed sizes and PDI values much lower than those obtained using thin film hydration or the ethanol injection method to generate transfersomes [40].

Stability studies showed high stability over time for the three formulations. However, different in vitro release patterns of MTM were observed for the different nanocarriers at pH 7.4. MTM-NPs showed an exponential release profile with an initial burst release no higher than 15%. MTM-NPs sustained a drug release profile over time, achieving the release of the MTM driven by diffusion after 24 h, in contrast with MTM-LIP whose release was slowed down after 8 h. The release of the MTM from MTM-HG was very fast, with a full release of MTM after 30 min. This burst release from HG has been previously described and may be better controlled through the combination of HG with other polymeric materials [41]. In any case, those nanoparticles showing more rapid release profiles, such as HG, may be suitable for local administration, while those nanodelivery systems with more controlled release, such as MTM-NPs or MTM-LIP, may be appropriate for systemic treatments [41].

In this work, we assayed the ability of our MTM nano-delivery systems to target different models of myxoid liposarcoma, undifferentiated pleomorphic sarcoma and chondrosarcoma. We show that all MTM-loaded nanocarriers retain the ability of free MTM to inhibit proliferation and invasion at nanomolar concentrations. As proof in favor of the safe use of these devices, we also show that the empty nanocarriers did not show any toxicity at any assayed concentration. More relevantly, we found that in vivo administration of MTM entrapped in liposomes resulted in a highly effective and dose-dependent antitumour response of sarcoma xenografts, including slight regression after the treatment with a dose of 2 mg/Kg MTM-LIP. Importantly, encapsulated MTM exhibited significantly enhanced therapeutic effects on these xenografts as compared with free MTM. Moreover, in vivo nanodelivery of MTM is able to highly reduce the toxicity effects of this drug at concentrations that demonstrated high antitumour activity against sarcomas. In line with these results, MTM encapsulated in two different types of polymeric nanoparticles, PLGA and poly-ursolic acid, also demonstrated higher anti-tumour activity than free MTM in a pancreatic cancer model and a colon cancer model respectively [17, 42]. Nevertheless, the doses of free MTM assayed in these works was not sufficient to produce histological damage, therefore our study is the first demonstrating a safer profile for encapsulated MTM.

A relevant antitumour feature of MTM is the ability to downregulate the expression of genes associated with the CSC phenotype and to target CSC subpopulations, as reported for glioblastoma [28], medulloblastoma [29], cervical cancer [26] and colon cancer [27]. Here we assessed the effect of MTM in stemness using two different functional methods associated to the clonogenic potential of tumour cells (tumoursphere growth) and to transcriptional activity of pluripotency factors (SORE6 activity). Our results expand the spectrum of tumours responding to the anti-stemness activity of MTM to sarcomas and also confirm that the MTM-delivering devices described here keep the anti-stemness potential of free MTM. This ability of MTM to target CSCs in sarcomas is in line with our previous work showing that the MTM analogue EC-8042 was able to inhibit CSC-associated gene expression in the same models used in this work and to target CSCs most efficiently than other drugs used to treat sarcomas [12, 25].

The effects of encapsulated MTM on gene expression had not been reported yet. In agreement with the similar in vitro antitumour activity of free and encapsulated MTM, RNA sequencing analyses showed that myxoid liposarcoma cells treated with either MTM-NPs or free MTM underwent a similar transcriptomic dysregulation process. In both cases, we observed a robust pattern of transcriptional repression leading to the inhibition of relevant cancer-related pathways like ECM receptor interaction, focal adhesion, mTOR signaling, VEGF signaling, ERBB signaling, TGFβ signaling or WNT signalling. This pattern of gene expression inhibition is in line with the ability of MTM to bind GC-rich sequences of the DNA, thus preventing the access of relevant transcription factor to their promoters and blocking the expression of downstream targets. Among those transcription factors inhibited by MTM, SP1 is the most widely described [6, 38, 43]. Indeed, we found that the treatment of sarcoma cells with both free and encapsulated MTM resulted in the inhibition of a wide array of SP1 downstream targets. Relevantly, many of these SP1-regulated factors, including VEGFA, TFGB, mTOR, EGFR, cMYC, ABCC1 or ITGA5, are key factors of the signalling pathways above described to be the most deeply inhibited by MTM. Among other factors commonly dowregulated after treatment with MTM or MTM-NPs (Additional files 5, 6: Tables S1 and S2), NTKR3 is known to be part of different oncogenic gene fusions in sarcomas and is being investigated as potential therapeutic target [44, 45]. Besides, CDH4 has been recently found to be overexpressed and to play key pro-tumour roles in osteosarcoma [46]. Altogether, these data suggest that MTM may act as multi-repressor agent able to inhibit the activation of several tumour-promoting pathways at once.

## Conclusions

The use of MTM delivery systems may offer a wide range of possibilities to overcome the multiple physiological barriers that must be overcome by MTM and improve its safety, specificity and efficiency. The results from this work confirm that MTM encapsulated in polymeric nanoparticles, liposomal formulations and hydrogels retain the ability of free MTM to repress the expression of relevant cancer-related pathways in sarcoma cells while improving its therapeutic efficacy and safety in vivo. Therefore, these novel formulations may represent an efficient and safer MTM-delivering alternative for sarcoma treatment.

## Methods

### Materials

PLA was synthesised under nitrogen using standard Schlenk techniques by ring-opening polymerisation using zinc alkyl as an initiator [47]. *Rac*-lactide was purchased from Sigma-Aldrich (Spain), sublimated three times, and stored in a glovebox. Toluene was pre-dried over sodium wire and distilled under nitrogen from sodium. Deuterated solvents were stored over activated 4 Å molecular sieves and degassed by several freeze-thaw cycles. Zinc compound was prepared according to literature procedures [48]. The solvents acetone (ACS reagent), toluene (ACS reagent) and tetrahydrofuran (inhibitor-free, for HPLC, ≥ 99.9%) were purchased from Sigma-Aldrich (Spain). Poly(vinyl alcohol) (PVA, 31,000–50,000, 98–99% hydrolysed) were purchased from Sigma-Aldrich (Spain) and used as received. L-α-Phosphatidylcholine (FOS) and Pluronic F-127 were purchased from Sigma-Aldrich (Spain), Miglyol-812 (MCFT) were purchased from Fagron Iberica.

Mithramycin (MTM) was synthesized by EntreChem SL (Oviedo, Spain) by a previously described combinatorial biosynthesis method [49]. Drug stocks were prepared as 10 mM solutions in sterile DMSO, maintained at −20 °C, and brought to the final concentration just before use.

### Gelation procedure and encapsulation methodology of MTM

(5-(3-(4-nitrophenyl)ureido)isophthalic acid) (hydrogelator) were obtained by slow pH change generated by the well-controlled hydrolysis of glucono-δ-lactone (GL) [30]. In a passive loading methodology 10 mg of hydrogelator were dissolved in 1 mL of 0.1 M aqueous NaOH. 1 mL of an aqueous solution of MTM (3 mg/mL) was added on the previous solution along with 2.5 mg of GL. The resulting solution was stored for 24 h for gelation. The formation of the composite gel was confirmed macroscopically by the observation of no gravitational flow upon the test tube inversion method.

### Liposomal formulation of MTM

Free cholesterol liposomes were formulated by ethanol injection and evaporation solvent method [32, 33]. Briefly, 20 mg of FOS and 70 µl of MCFT were dissolved in 250 µl of ethanol. 3 mg of MTM were dissolved in 4.75 mL of acetone and were mixed with lipids to form the organic phase. The organic phase was added dropwise over Pluronic F-127 solution (1 mg/mL) under vigorous stirring and homogenized 10 min at 14,000 rpm. The organic solvent was evaporated under reduced pressure at 40 °C for 20 min. The liposomes suspension was dialyzed against mQ water for 1 h at 25 °C in a 3500 MWCO dialysis cassette to eliminate the excess of surfactant and unencapsulated MTM. The mQ water after dialysis was used to calculate the encapsulation efficiency and loading efficiency of formulations.

### Polymeric nanoparticles formulation

MTM loaded NPs (MTM-NPs) were formulated by nanoprecipitation and displacement solvent method [31]. Briefly, 80 mg of PLA and 10 mg of MTM were dissolved in 6 mL of acetone to form the organic phase. Then, the organic phase was added dropwise over 10 mL of PVA solution (1%) with a flowrate of 2 ml/min under continuous stirring. The acetone was evaporated under reduced pressure to form the NPs suspension. After centrifugation at 15,000 rpm for 20 min, the MTM-NPs were collected and washes several times to eliminate unencapsulated MTM and excess of surfactant. The MTM-NPs were resuspended in mQ water for subsequent freeze drying and stored at −20 °C to use in all other experiments.

### Characterization of formulations

SEM images were recorded on a Jeol 6490LV electron microscope to evaluate the size and morphology of the particles. High resolution electron microscope images were acquired on a Jeol JEM 2100 TEM microscope operating at 200 kV and equipped with an Oxford Link EDS detector. As the specimens were sensitive to beam irradiation, observation was performed under low-dose conditions. The resulting images were analyzed using Digital Micrograph™ software from Gatan. The average sizes, polydispersities and Z-potentials of the formulations were measured using a Zetasizer Nano ZS (Malvern Instruments) Data were analysed using the multimodal number distribution software included with the instrument.

### Efficiency and loading efficiencies

To calculate the loading efficiency and encapsulation efficiency for MTM-NPs, 1 mL of DMSO was added to NPs to dissolve both polymer and drug and the mixture was subjected to bath sonication for 30 min. MTM loading was measured in a spectrophotometer at 570 nm of wavelength.

Loading efficiency of liposomes and encapsulation efficiency were calculated by using the difference of MTM feeding and the non-encapsulated MTM found in the supernatant after dialysis in phosphate buffered saline medium.

LE and EE of MTM were calculated according to the following equations:

LE% = (weight of encapsulated MTM (mg))/(weight of total (MTM encapsulated + scaffold weight) (mg)) × 100%.

EE% = (weight of encapsulated MTM (mg))/(weight of MTM feeding (mg)) × 100%.

### Release studies

*MTM-loaded NPs.* 10 mg of lyophilized MTM-NPs were placed in dialysis membrane (molecular weight cut off: 3500 KDA) and incubated in 10 mL of Phosphate Buffered-Saline (PBS, pH 7.4). The suspension was incubated at 37 °C with continuous stirring (50 rpm) in a IKA incubator shaker KS 3000. At different intervals of incubation 3 mL of release medium was removed to measure fluorescence emission in a fluorimeter at 570 nm of wavelength.

*MTM-loaded LIP.*1 mL of liposomes suspension were released in the same method described above.

*MTM-loaded HG.* In a spectrophotometer cuvette were placed 0.25 mL of MTM-HG and 2.75 mL of PBS (pH 7.4) and were incubated at 37 °C. At different intervals of incubation, the amount of MTM released to PBS were measured in a fluorimeter at 570 nm of wavelength.

### Stability studies

The NPs were incubated at 37 °C (1 mg·mL^− 1^) in PBS and Liposomes were incubated at 37 °C resuspending 100 µL of liposomes in 1 mL of PBS. The average size (nm) and PdI were determined over time by DLS measurements. The stability of the MTM-LIP was also performed in 10% human blood plasma (obtained from the Blood Donor Center of the University Hospital of Albacete, CHUA, Spain). MTM-LIP were incubated at 37 °C, at a concentration equal to 1 mg·mL^− 1^. The hydrodynamic radius (RH) of the formulation was calculated at predetermined intervals of time by DLS measurements.

### Cell models

The myxoid liposarcoma model MSC-5 H-FC cells were generated upon sequential mutation of human bone marrow MSCs with up to 6 oncogenic events: (i) hTERT overexpression; (ii) P53 and (iii) Rb inactivation using E6 and E7 antigens of the HPV-16; (iv) inactivation of PPA2 phosphatase with SV40 small T antigen; (v) expression of oncogenic H-RASv − 12; and (vi) expression of fusion oncogene FUS-CHOP (FC) [50–52]. T-5 H-FC#1 is a cell line derived from a xenograft tumour generated by MSC-5 H-FC cells and represent a more aggressive myxoid liposarcoma model than its parental cell line [35, 52]. The related undifferentiated pleomorphic sarcoma model T-5 H-O cells (which were targeted with above described mutations i) to v) [35]) expressing the SORE6 lentiviral reporter system (T-5 H-O-SORE6-GFP cells) or its negative gating control (T-5 H-O-minCMV-GFP cells) were previously generated and characterized [25]. T-CDS-17#4 is a cell line derived from a xenograft generated by the patient-derived chrondrosarcoma primary cell line CDS-17, which have been comprehensively characterized at genomic and functional level elsewhere [53]. The identity of these cell lines has been authenticated by a Short Tandem Repeats analysis during the last 5 months. All the cell types were cultured as previously described [12, 53].

### Cell viability assays

The viability of cell lines in the presence and absence of drugs was determined using the cell proliferation reagent WST-1 (Roche, Mannheim, Germany) after 72 h-treatments as described before [12]. The concentration of half-maximal inhibition of viability (IC_50_) for each treatment was determined by non-linear regression using GraphPad Prism version 8.0 (Graphpad Software Inc, La Jolla, CA). Alternatively, the cytotoxic potential of the assayed treatments was assayed in colony formation unit (CFU) assays as described previously [54]. In these assays, cells were treated for 24 h and left to form colonies in drug-free medium for 10 days. The surviving fraction was determined by dividing the average number of colonies for each treatment by the average number of colonies in the control. For the administration of MTM-HG, vortex occasionally before incubation is needed prior to handle the sample with a syringe. Then, MTM-HG is subsequently added to each well and the cells are incubated as described.

### Tumoursphere culture

Tumoursphere formation protocol and the analysis of the effects of drugs on tumoursphere formation ability were previously described [12, 55].

### Three-dimensional spheroid invasion assay

Cell spheroids were prepared using a hanging drop protocol as previously described [56]. Then each cell spheroid was transferred to an individual well of 96-well plate and embedded into a volume of 70 µl of 3 mg/ml bovine collagen type I matrix (PureCol) from Advanced Biomatrix (San Diego, CA) and filled with 100 µl of complete media. Cell invasion in the presence or not of free or encapsulated MTM was monitored using a Zeiss Cell Observer Live Imaging microscope (Zeiss, Thornwood, NY) and images were acquired every 6 h during 24 h using a Zeiss AxioCam MRc camera. The invasive area was determined by calculating the difference between the final area (t = 24 h) and the initial area (t = 0 h) using the Image J analysis software, and data were normalized to the control cells. 3 independent experiments including 4 replicates for each condition were performed.

### Detection of SORE6 activity by flow cytometry

The lentiviral reporter systems in which a SORE6 element coupled to a minimal cytomegalovirus (mCMV) drive the expression of GFP (SORE6-mCMVp-dsCopGFP-Puro) and the corresponding control lacking SORE6 (mCMVp-dsCopGFP-Puro) were previously generated and characterized [36] and kindly donated by Dr. L.M. Wakefield (National Cancer Institute, Bethesda, MD). Sarcoma cells expressing the SORE6 system (T-5 H-O-SORE6-GFP cells) or its negative control (T-5 H-O-minCMV-GFP cells) were previously generated and described [25]. The level of SORE6-driven GFP fluorescence in untreated cultures or after different drug treatments were analyzed by flow cytometry using a BD FACS Aria II Cell Sorter (BD Bioscience, Erembodegem, Belgium). Control T-5 H-O-minCMV-GFP cells were used as matched SORE6 negative control for gating purposes.

### Western blotting

Whole cell protein extraction and Western blot analysis were performed as previously described [57]. Primary antibodies used in these analyses were as follow: anti-SP1 [(9389), 1:1000 dilution] and anti-Survivin [(2808), 1:1000] from Cell Signaling (Danvers, MA); anti-c-MYC [(sc-40), 1:100], anti-VEGF [(sc-57,496), 1:100] and anti-Bcl-2 [(sc-783), 1:100] from Santa Cruz Biotechnology (Dallas, TX); and anti-β-Actin [(A5441), 1:10,000] from Sigma. Detection and quantification of the protein bands (IRDye fluorescent signals) was performed using the Odyssey Fc imaging system and the software Image Studio from LICOR (Lincoln, NE).

### RNA seq

High-quality RNA samples were used to prepare RNA-seq libraries according to NEBNext® Ultra Directional RNA library protocol (New England Biolabs, Ipswich, MA). cDNA libraries were checked for quality and quantified using the DNA-1000 kit (Agilent) on a 2100 Bioanalyzer. Paired-end sequencing was performed on an Illumina Novaseq 6000 (Illumina, San Diego, CA, USA) using 150-base reads. Then pseudo-aligment and quantification of transcripts were made with Salmon algorithm (reference genome GRCh38). Correlation analysis, principal component study and differential expression analysis were performed with DESeq2 package. Differential gene expression analysis was done using the parametric Wald test with Benjamini-Hochberg adjustment (padj). Genes with padj < 0.01 and (fold change ≤-−2 or ≥ 2) were considered significantly expressed genes. FGSEA package (http://bioconductor.org/packages/release/bioc/html/fgsea.html) was used for gene set enrichment analysis of KEGG pathways and GO terms (Cellular Component–Biological Processes–Molecular Function).

### Treatment of tumour xenografts

All experimental protocols were carried out in accordance with the institutional guidelines of the University of Oviedo and were approved by the Animal Research Ethical Committee of the University of Oviedo. Female athymic nude mice of 6 weeks old (Envigo, Barcelona, Spain) were inoculated subcutaneously (s.c.) with 5 × 10^5^ T5H-FC#1 cells. Once tumours reached approximately 100 mm^3^, the mice were randomly assigned (n = 5 per group) to receive the following intravenous treatments twice a week (6 doses): vehicle (PBS), 1 mg/Kg MTM, 2 mg/Kg MTM, 1 mg/kg MTM-LIP or 2 mg/Kg MTM-LIP. Mean tumour volume differences between groups were determined using a caliper as described [58]. Relative tumour volume (RTV) for every xenograft was calculated as follows: RTV = tumour volume at day of measurement (V_t_)−tumour volume at the beginning of the treatment (V_o_). Drug efficacy was expressed as the percentage tumour growth inhibition (%TGI), calculated using the Eq. 100-(T/C×100), where T is mean RTV of the treated tumour and C is the mean RTV in the control group at day of measurement. Animals were sacrificed by CO_2_ asphyxiation when tumours of the control series reached approximately 1000 mm^3^. Livers were extracted and processed for histological analysis as previously described [59].

### Statistical analyses

Statistical analysis was performed using GraphPad Prism version 8.0 (Graphpad Software Inc, La Jolla, CA, USA). Data are presented as the mean (± standard deviation or SEM as indicated) of at least three independent experiments. Two-sided Student’s t test was performed to determine the statistical significance between groups. Multiple comparisons of the data were performed using the one-way ANOVA, Turkey’s test. p < 0.05 values were considered statistically significant.

## Supplementary Information

**Additional file 1: Fig. S1.** Stability of MTM-LIP nanovesicles in human blood plasma.

**Additional file 2: Fig. S2.** Antiproliferative effects induced by encapsulated MTM and empty nanoparticles in myxoid liposarcoma models.

**Additional file 3: Fig. S3.** Invasion ability of free and encapsulated MTM-treated sarcoma cells.

**Additional file 4: Fig. S4.** Gating strategy used to analyze SORE6-GFP activity.

**Additional file 5: Table S1.** RNA-seq analysis showing genes up- and down-regulated in cells treated with free MTM vs control.

**Additional file 6: Table S2.** RNA-seq analysis showing genes up- and down-regulated in cells treated with MTM-NPs vs control.

**Additional file 7: Table S3.** RNA-seq analysis showing KEGG-pathways up- and down-regulated in cells treated with free MTM vs control.

**Additional file 8: Table S4.** RNA-seq analysis showing KEGG-pathways up- and down-regulated in cells treated with free MTM vs control.

**Additional file 9: Table S5.** RNA-seq analysis showing KEGG-pathways up- and down-regulated in cells treated with MTM-NPs vs control.

## Data Availability

The RNA seq datasets generated during the study are available in the GEO-NCBI repository (Reference: GSE161616; https://www.ncbi.nlm.nih.gov/geo/query/acc.cgi?acc=GSE161616).

## Abbreviations

CSCs: Cancer stem cells
CFU: Colony formation unit
mCMV: Cytomegalovirus
DEG: Differentially expressed genes
DLS: Dynamic light scattering
EE: Encapsulation efficiency
FC: FUS-CHOP
GSEA: Gene Set Enrichment Analysis
GL: Glucono-δ-lactone
R_H_: Hydrodynamic radius
HG: Hydrogels
LIP: Liposomes
LE: Loading efficiency
FOS: L-α-Phosphatidylcholine
MSCs: Mesenchymal stromal/stem cells
MCFT: Miglyol-812
MTM: Mithramycin
NPs: Nanoparticles
PLGA: Poly(lactic-co-glycolic acid
PLA: Poly(rac-lactide)/polylactide
PVA: Poly(vinyl alcohol)
PdI: Polydispersity index
PC: Principal component
SEM: Scanning Electron Microscopy
SORE6: SOX2/OCT4 response element
TEM: Transmission electron microscopy
