# Supplementary figures and images for: Mithramycin delivery systems to develop effective therapies in sarcomas
Source: J Nanobiotechnology. 2021 Sep 6;19:267. doi: 10.1186/s12951-021-01008-x (PMC8419920; doi:10.1186/s12951-021-01008-x)

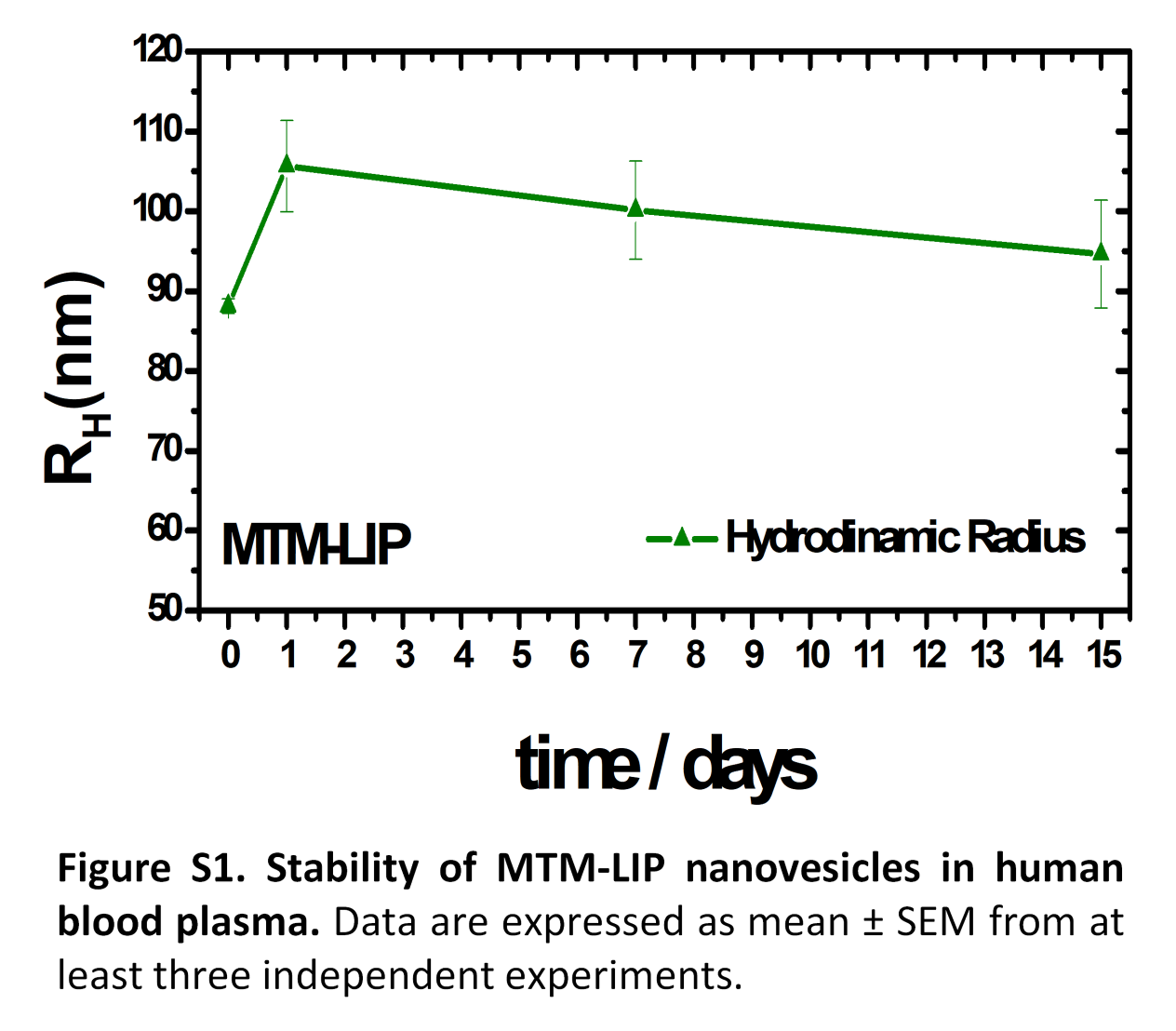

Supplement: Supplementary file 1 — Additional file 1: Fig. S1. Stability of MTM-LIP nanovesicles in human blood plasma. [file 12951_2021_1008_MOESM1_ESM.tif]

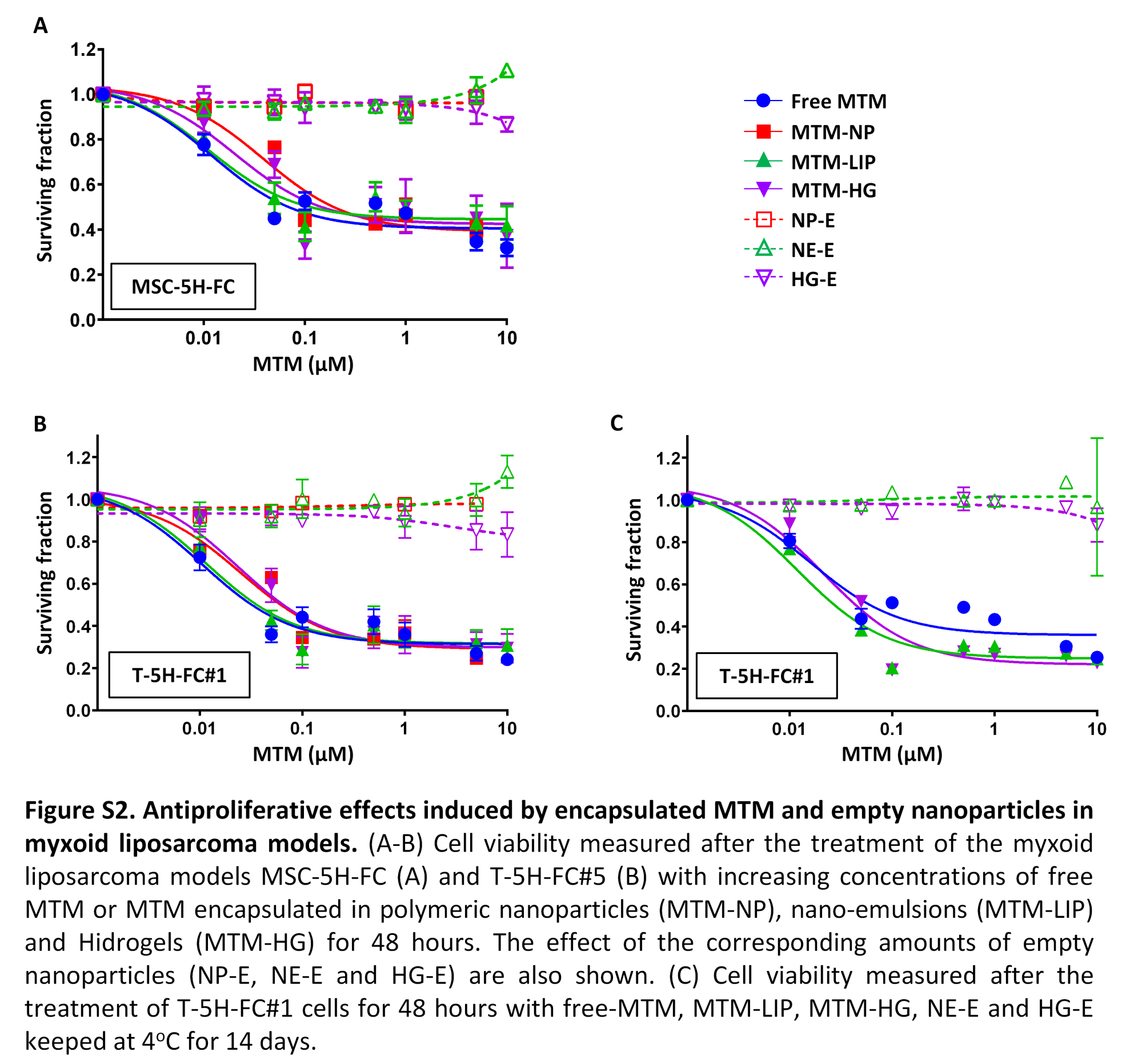

Supplement: Supplementary file 2 — Additional file 2: Fig. S2. Antiproliferative effects induced by encapsulated MTM and empty nanoparticles in myxoid liposarcoma models. [file 12951_2021_1008_MOESM2_ESM.tif]

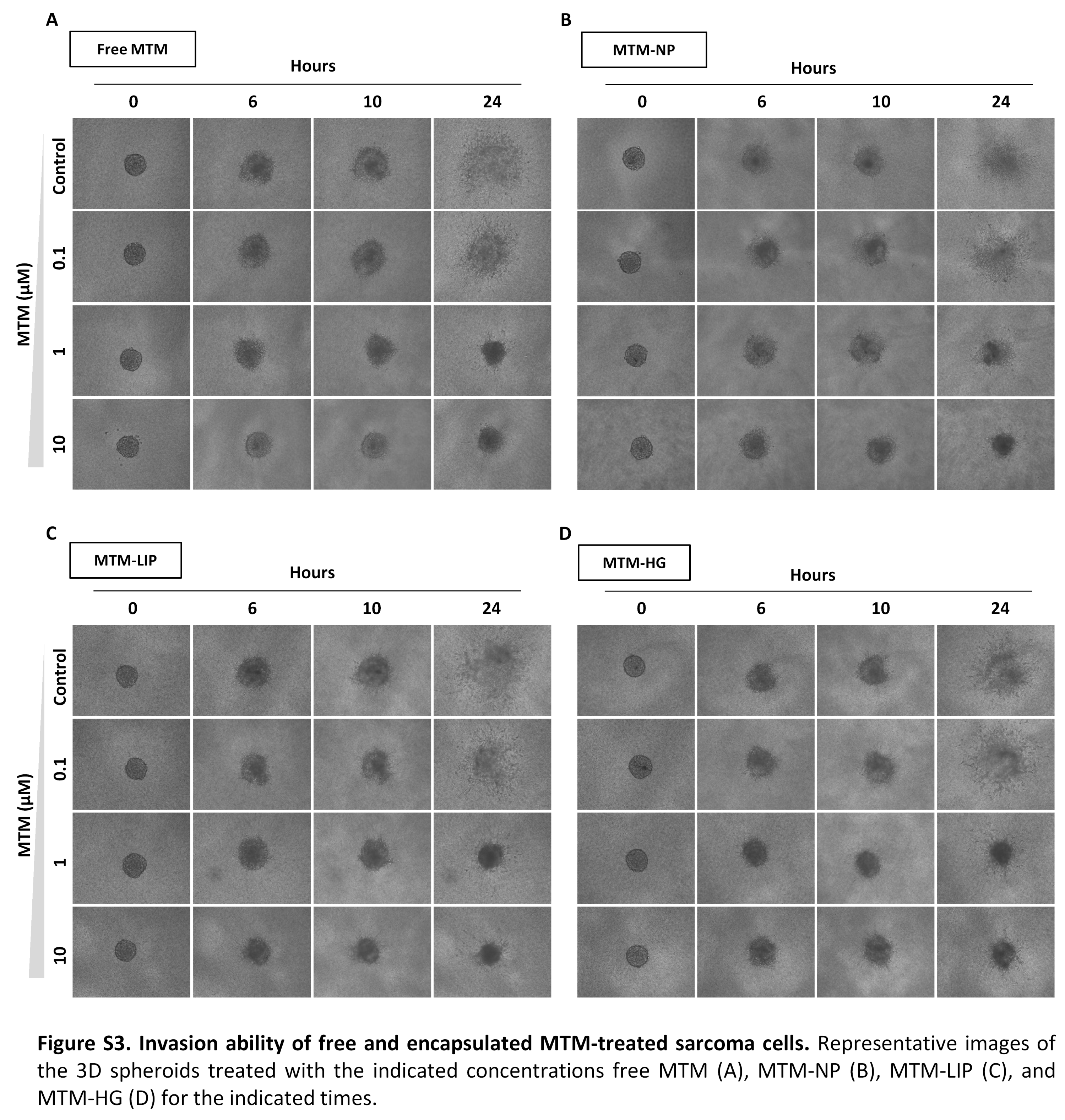

Supplement: Supplementary file 3 — Additional file 3: Fig. S3. Invasion ability of free and encapsulated MTM-treated sarcoma cells. [file 12951_2021_1008_MOESM3_ESM.tif]

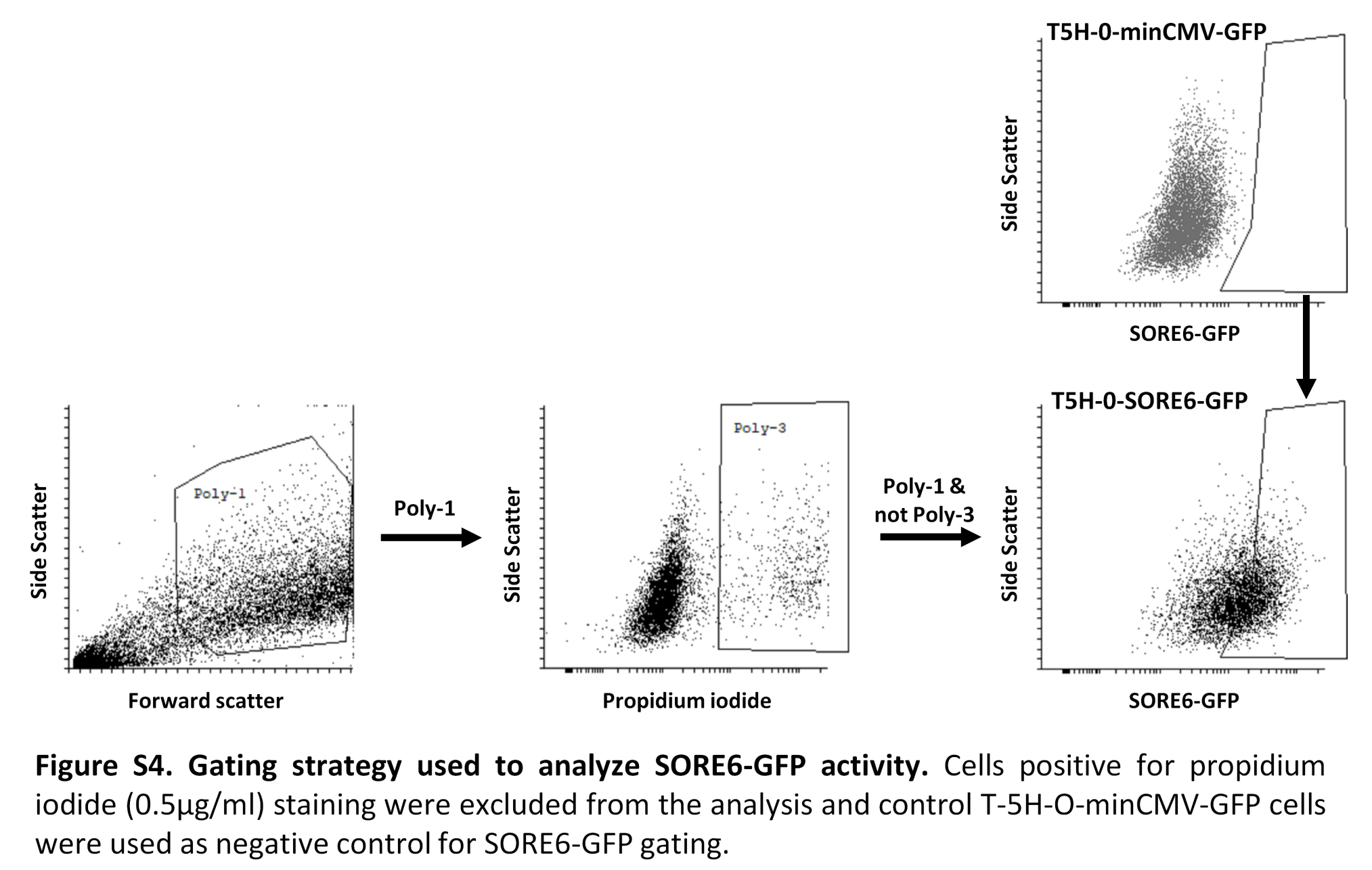

Supplement: Supplementary file 4 — Additional file 4: Fig. S4. Gating strategy used to analyze SORE6-GFP activity. [file 12951_2021_1008_MOESM4_ESM.tif]
